# Review on the structural understanding of the 10S myosin II in the era of Cryo-electron microscopy

**DOI:** 10.1186/s42649-022-00078-x

**Published:** 2022-10-11

**Authors:** Anahita Vispi Bharda, Hyun Suk Jung

**Affiliations:** Division of Chemistry & Biochemistry, Department of Biochemistry, College of Natural Sciences, Kangwon National University, Chuncheon, Gangwon 24341 Republic of Korea

**Keywords:** Smooth muscle myosin-II, Myosin, Cryo-electron microscopy, Structural biology, High resolution studies, Molecular motor

## Abstract

The compact smooth muscle 10S myosin II is a type of a monomer with folded tail and the heads bending back to interact with each other. This inactivated form is associated with regulatory and enzymatic activities affecting myosin processivity with actin filaments as well as ATPase activity. Phosphorylation by RLC can however, shuttle myosin from the inhibited 10S state to an activated 6S state, dictating the equilibrium. Multiple studies contributed by TEM have provided insights in the structural understanding of the 10S form. However, it is only recently that the true potential of Cryo-EM in deciphering the intramolecular interactions of 10S myosin state has been realized. This has led to an influx of new revelations on the 10S inactivation, unfolding mechanism and association in various diseases. This study reviews the gradual development in the structural interpretation of 10S species from TEM to Cryo-EM era. Furthermore, we discuss the utility of Cryo-EM in future myosin 10S studies and its contribution to human health.

## Introduction

**Fig. 1:**
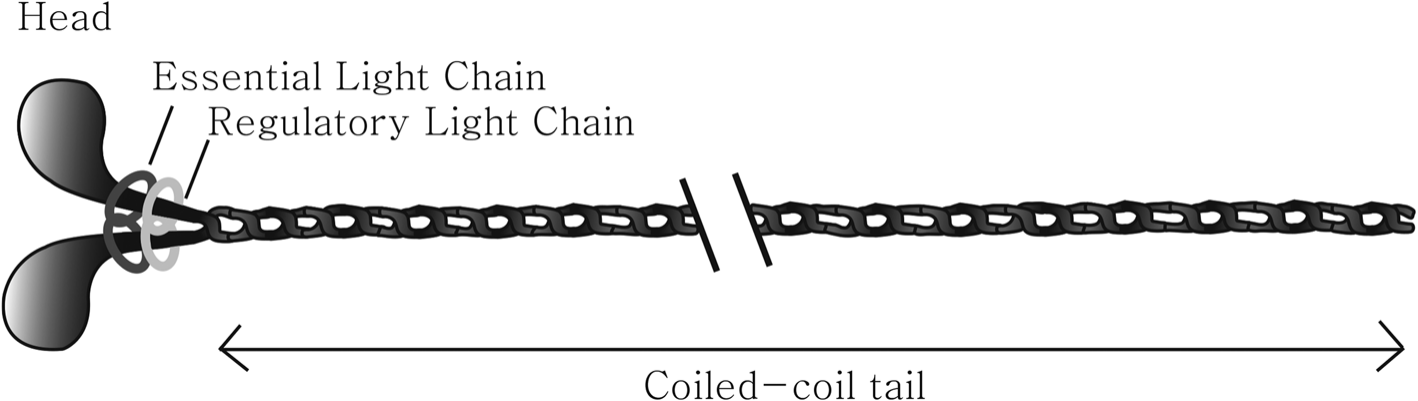
Schematic representation of a typical smooth muscle myosin II molecule. Myosin II is composed of two heavy chains (HCs), two essential light chains (ELCs) and two regulatory light chains (RLCs) to form a 520 kDa protein. The N-terminal forms the globular heads and C-terminal extends as a long α-helical coiled-coil tail

Myosin II is a conventional molecular motor protein that moves actively along the actin filament through a force driven by ATP hydrolysis (Geeves and Holmes 1999). A myriad of studies have reported multifaceted roles of myosin in both muscles and non-muscles (Conti and Adelstein 2008; Vicente-Manzanares et al. 2009; Ma and Adelstein 2014; Shutova and Svitkina 2018). Typically, myosin molecule possesses two identical heavy chains, a pair of essential light chains (ELCs) and a pair of regulatory light chains (RLCs) as seen in Fig. 1. The amino-terminal of each heavy chain encompasses a globular head domain that harbours the ATP and actin binding sites and a neck domain where the light chains reside. The carboxy-terminal is primarily a long alpha-helical coiled-coil tail domain which is associated with dimerization (Rayment et al. 1993). In smooth muscles, this tail helps myosin molecules associate together to form side-polar filaments and perform various functions (Craig and Woodhead 2006). Studies of proteolytic digestion of myosin molecule show 2 major subfragments: Heavy meromyosin (HMM) spanning myosin subfragment 1 (S1) and myosin subfragment 2 (S2) and light meromyosin (LMM) including the tail backbone.

Myosin II monomers can exist in two different conformations based on their sedimentation coefficients – an open 6S conformation and a closed 10S conformation (Suzuki et al. 1978). The closed 10S conformers were found to be present in both smooth muscle and non-muscle systems (Trybus et al. 1982; Milton et al. 2011). Myosin in the 10S conformation is inactive and forms weak interaction with actin (Olney et al. 1996). Structurally, the 10S conformation involves an equidistant, three segmented tail folding where the heads lean back on to the tail to associate with each other (Trybus et al. 1982; Burgess et al. 2007; Jung et al. 2008b). This interaction, while being very specific, occurs in an asymmetric fashion and is highly conserved across species (Wendt et al. 2001; Jung et al. 2008a). Within the twin head interaction, by-products of ATP turnover are sequestered affecting the ATPase activity (Cross et al. 1986; Cross et al. 1988; Ankrett et al. 1991). As a consequence, myosin monomers cannot unfold to polymerize into filaments nor can they interact with actin, achieving a total shut off state. Conversely, RLC phosphorylation at Ser19 residue destabilizes the 10S molecule causing the tail to unfold (Craig et al. 1983; Trybus and Lowey 1984). This creates a shift in the dynamics from 10S monomers to the extended 6S molecules facilitating filament assembly. At molecular level, RLC phosphorylation in smooth muscles occurs through Ca^2+^/calmodulin dependent myosin light chain kinase which is exclusively tissue specific (Sellers 1991).

The functional significance of the 10S regulation traces back to the origin of animals with head-head and head-tail interactions conserved all throughout (Lee et al. 2018). This shut-off state plays a pivotal role in maintaining the energy equilibrium when myosin activity is not in demand for muscle as well as non-muscle (Cross 1988). Much of these findings was possible with significant development in the electron microscopic approaches. Electron microscopy has played a defining role in understanding the structure and function of myosin over the last six decades (Hanson and Huxley 1953; Huxley 1957).

## Preliminary developments in the structural biology of myosin II

Contribution of structure determining techniques like metal shadowing and negative staining - TEM as well as X-ray diffraction has led to phenomenal growth in the field of myosin biology. Early TEM studies documented 10S as a dimer (Suzuki et al. 1978) which later was found to be a monomer (Trybus et al. 1982). Addition of ATP in stoichiometric amount, depolymerized the smooth muscle filaments to give inactivated monomers (Suzuki et al. 1978; Suzuki et al. 1982). When observed with rotary shadowing, 10S monomers were predominantly found under low salt concentration in a folded conformation (Trybus et al. 1982). The arrangement of the tail was in such a manner that it folded as a 510 Å hairpin loop with the two heads leaning over (Suzuki et al. 1982). The heads went on to interact with each other in an asymmetric manner to perturb their activities. This association, also termed as the interacting-heads motif or IHM is due to intramolecular interaction occurring at both, “blocked” and “free” heads. The inhibition occurs so that each head is responsible for hampering the other head’s activity. As a result, actin binding ability of the blocked head and ATPase activity of the free head is affected causing a total switch off. While the first report of IHM was performed on smooth muscle HMM through X-ray diffraction (Wendt et al. 2001), evolutionary analysis revealed myosin 10S to be conserved since the origin of animals (Lee et al. 2018). These studies clearly indicated IHM as a structurally important feature in the functionality of 10S myosin.

Another striking feature of the 10S monomer is the propensity of the tail to fold at two particular locations to give three segments of equivalent length (Trybus et al. 1982; Wendt et al. 2001; Burgess et al. 2007). Each segment spanned a region of its own, running between and around the two heads generating head-tail interactions (Jung et al. 2008b). Primitive TEM approaches including metal shadowing feared the possibility of structural rearrangements (Trybus and Lowey 1984), however adapting cross-linkers like glutaraldehyde in small concentrations proved beneficial in negative staining TEM (Jung et al. 2011). The cross-linking mechanism was non-specific and provided stability to the compact structures. Moreover, technical advances in CCD cameras and image processing software made it feasible to understand the stereospecific interactions occurring within the monomer. Nevertheless, myosin 10S structures provided by TEM with single particle analysis reached a maximum resolution limit of ~ 20 A°.

## Revolution in myosin structural studies through Cryo-Electron microscopy

For many years, battle over atomic resolution model of proteins remained in the favour of X-ray crystallography, only until Dubochet and his colleagues established Cryo-electron microscopy (Cryo-EM) (Dubochet et al. 1988). Their pioneering work led to a dramatic change in the way biological samples could be applied for electron microscopic examination. The procedure involved vitrification, which enabled samples to be embedded in a thin layer of aqueous solution followed by rapid plunge-freezing in liquid ethane, capturing the sample in its native state. Cryo-EM paved the way for modern TEM with crucial developments in detection cameras and Low-dose electron exposure. This caused relatively lower radiation damage to biological samples and eliminated the need to prepare high concentration sample for crystallization. Interdisciplinary approach using Cryo-EM with advanced computational tools gave momentum to 3D structural determination reaching near atomic resolution. Looking at the phenomenal progress, Nobel prize in Chemistry was granted to Jacques Dubochet, Richard Henderson and Joachim Frank together in 2017 (Cressey and Callaway 2017) for developing Cryo-EM.

Recently, Cryo-EM reports on 10S have articulated crucial insights on the activation, regulation of Myosin 10S (Scarff et al. 2020; Yang et al. 2020). Some of the major findings disclosed were comparable with regions previously discovered by X-ray crystallography. With Cryo-EM, it was now possible to understand the intricacies of the 10S molecules with high resolution. Moreover, these studies served as the base to delve deeper in to the different types of molecular interactions that made up the 10S state. Some of the striking features included: The tail showing great degree of flexibility in 10S molecule, with multiple head and tail interactions. These interactions formed the basis of stability of the molecule affecting the ATP binding moiety of the free head and the actin binding region in both heads. The probable mechanism for destabilization of the shut-down 10S form was emphasized on RLC phosphorylation. Since the structural densities corresponding to the RLC of both free and blocked head were disproportional, RLC interactions lead to different consequences. Depression in the ATPase activity of the blocked head was well defined with the predicted interaction between tail segment (Segment 2) and the blocked head (Yang et al. 2020).

## Application of muscle structural studies in human health using Cryo-EM

During the last few years, Cryo-EM has set a new benchmark in the field of structural biology. A series of new revelations in myosin structural biology have been witnessed, which weren’t fully achieved before. For example, some interactions observed in the 10S structure propose similarity with the regions associated with disease causing mutations in smooth muscle and non-muscle myosin (Wendt et al. 2001; Scarff et al. 2020). Given that 10S is a highly conserved structure across multiple species, it is imperative to establish a better understanding in role 10S plays in multiple diseases. Interpretation from such interactions can provide leads on the mutations associated with cardiomyopathy.

## Conclusion and future perspectives

**Fig. 2:**
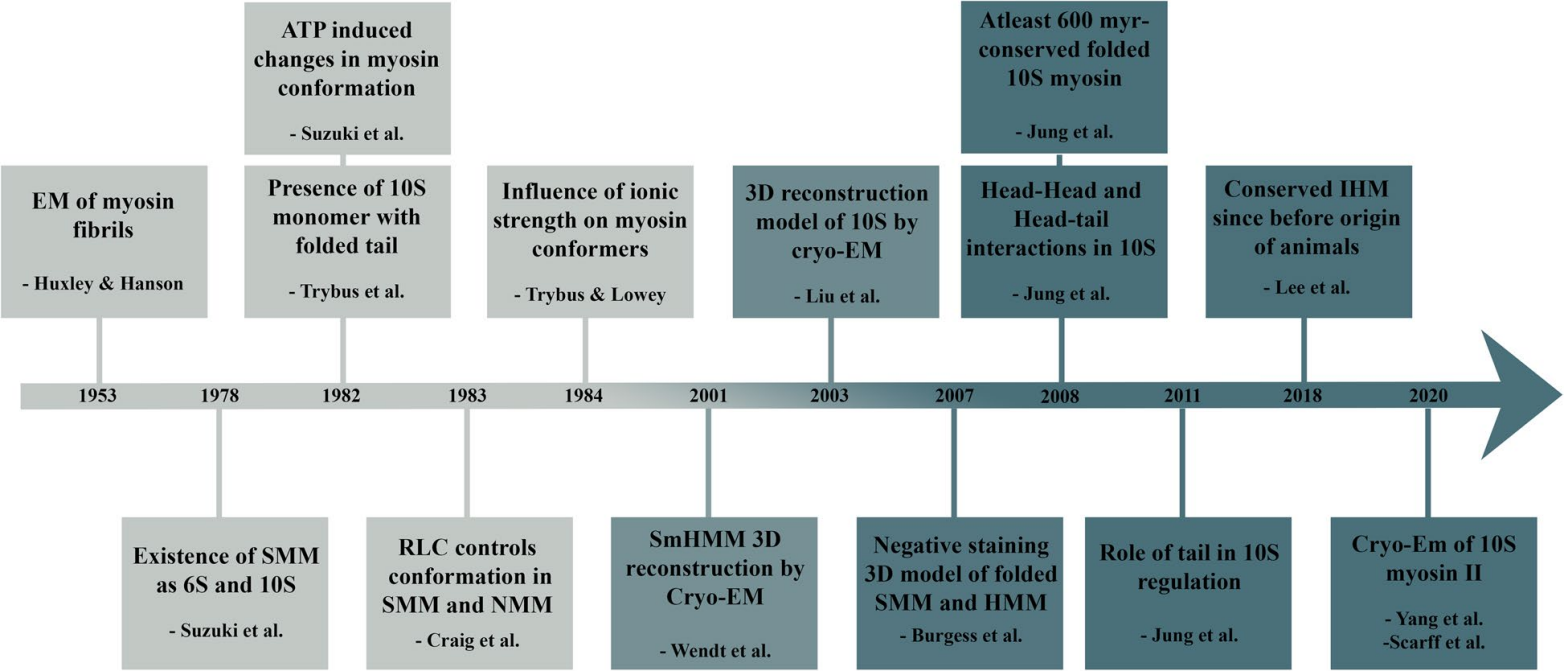
Conclusive illustration of the timeline for major events in the development of smooth muscle myosin 10S structural investigations

The functional and structural understanding of the 10S myosin II over last six decades has been summarized in Fig. 2. At present, Cryo-EM has offered remarkable upgrade in the field of myosin structural biology since the advent of conventional EM. Having said this, looking at the current developments made, it is likely that many trivial questions will be answered in the future. Clarity on the overall unfolding mechanism including the sequential order of the unfolding along with intermediate steps taking place at sub-molecular state will continue to be of focus in future. It is expected that research in the upcoming decades could transform from an in vitro to a more in vivo approach for real time application.

## Data Availability

All data generated or analyzed during this study are included in this article and no datasets were generated or analyzed during the current study.

## Abbreviations

6S: Sedimentation coefficients of 6
10S: Sedimentation coefficients of 10
ATP: Adenosine 5′-triphosphate
ATPase: Adenosine Tri-phosphatase
kDa: kiloDalton
Da: Dalton
SMM: Smooth muscle myosin
MLCK: Myosin Light Chain Kinase
ELC: Essential Light Chain
RLC: Regulatory Light Chain
Ca^2+^: Calcium cation
TEM: Transmission Electron Microscopy
EM: Electron microscopy (generalized for TEM here)
S1: myosin subfragment 1
S2: myosin subfragment 2
HMM: Heavy meromyosin
LMM: Light meromyosin
X-ray: X-radiation
Å: Angstrom
IHM: interacting-heads motif
CCD: Charge-couple device
Cryo-EM: Cryo-electron microscopy
3D: Three-Dimensional
myr: million year
